# Association of serum Krebs von den Lungen-6 and chest CT as potential prognostic factors in severe acute respiratory syndrome SARS-CoV-2: a preliminary experience

**DOI:** 10.1007/s11547-022-01504-6

**Published:** 2022-06-15

**Authors:** Emanuela Anastasi, Lucia Manganaro, Elisa Guiducci, Simone Ciaglia, Miriam Dolciami, Alessandra Spagnoli, Francesco Alessandri, Antonio Angeloni, Annarita Vestri, Carlo Catalano, Paolo Ricci

**Affiliations:** 1 Emergency Radiology Unit, Department of Diagnostic Medicine and Radiology, AOU Policlinico Umberto I, Sapienza University of Rome, Viale Regina Elena 324, 00161 Rome, Italy; 2 Department of Radiological, Oncological and Pathological Sciences, AOU Policlinico Umberto I, Sapienza University of Rome, Viale Regina Elena 324, 00161 Rome, Italy; 3 Department of Experimental Medicine, AOU Policlinico Umberto I, Sapienza University of Rome, Viale Regina Elena 324, 00161 Rome, Italy; 4 Department of Public Health and Infectious Diseases, AOU Policlinico Umberto I, Sapienza University of Rome, Viale Regina Elena 324, 00161 Rome, Italy; 5 Department of General and Specialist Surgery, AOU Policlinico Umberto I, Sapienza University of Rome, Viale Regina Elena 324, 00161 Rome, Italy

**Keywords:** COVID-19 pneumonia, CT semiquantitative score, KL-6, ARDS

## Abstract

**Purpose:**

To correlate in COVID-19 pneumonia CT-based semi-quantitative score of pulmonary involvement with high serum levels of KL-6, a biomarker of disease severity.

**Methods:**

Between March 28 to May 21, 2020, 196 patients with strong suspicion of SARS-CoV-2 were evaluated with RT-PCR for SARS-CoV-2, chest CT scan and blood test, including KL-6 serum protein, in our Emergency Unit. The final population included only patients who underwent blood sampling for KL-6 within 5 days from CT scan (*n* = 63), including *n* = 37 COVID-19-positive patients and *n* = 26 with negative RT-PCR testing for SARS-CoV-2 (control group). A semi-quantitative CT score was calculated based on the extent of lobar involvement (0:0%; 1, < 5%; 2:5–25%; 3:26–50%; 4:51–75%; 5, > 75%; range 0–5; global score 0–25).

**Results:**

CT score was significantly correlated with serum value of KL-6 (*r* = 27, *p* = 0.035). This correlation was also present in COVID-19 positive patients (*r* = 0.423, *p* = 0.009) and CT score median value was significantly higher in patients with high KL-6 value (> 400 U/mL; 12.00, IQR 5.00-18.00, p-value 0.027). In control group, no statistically significant correlation was found between CT score and KL-6 value and CT score was higher in patients with high KL-6, although this difference was not statistically significant (5.00, IQR:1.75–8.00 versus 3.50, IQR:2.00–6.50). "Crazy paving" at the right upper (*n* = 8; 61.5%) and middle lobe (*n* = 4; 30.8%) and "consolidation" at the middle lobe (n=5; 38.5%) were observed in COVID-19 group with a significant difference between patients with high KL-6 value.

**Conclusion:**

CT score is highly correlated with KL-6 value in COVID-19 patients and might be beneficial to speed-up diagnostic workflow in symptomatic cases.

## Introduction

New coronavirus pneumonia (COVID-19) has emerged since December 2019, and the World Health Organization declared a global health emergency on January 30, 2020 [1]. Nearly two years after the outbreak of the pandemic, there have been close to 430 million confirmed cases of COVID-19 worldwide, including more than 5.5 million deaths [2].

The most common clinical symptoms at presentation are fever and cough in addition to other nonspecific symptoms including dyspnea, headache, muscle aches and fatigue, as well as many non-pulmonary manifestations, such as diarrhea and muscle soreness and neurological impairment. Additionally, many patients have no clinical symptoms, this being one of the causes of the virus spreading [3–6].

Septic shock, acute respiratory distress syndrome, disseminated intravascular coagulation (DIC), and multi-organ failure are considered the most severe forms of this disease [7]. The case-fatality rate is higher among patients with preexisting disease, reaching about: 10.5% for cardiovascular disease, 7.3% for diabetes, 6.3% for chronic respiratory disease [8, 9].

Disease behavior is very heterogeneous and unpredictable, thus necessitating the identification of biomarkers that can assess the course of the disease. This purpose has been addressed in particular by laboratory biomarkers because of their relatively easy accessibility despite mainly obtaining nonspecific results. An increased D-dimer level is the most common laboratory finding in hospitalized patients with Sars-Cov2 infection [10]. Moreover, ferritin has been found to be drastically altered in COVID-19 patients with severe disease [11]. Several reports have also shown that vitamins D and K, which have so far been examined in neoplastic diseases, are dramatically altered in COVID-19 patients [12, 13].

What is emerging from recent literature is how the Krebs von den Lungen-6 (KL-6), a glycoprotein secreted by type II alveolar pneumocytes and bronchiolar epithelial, can be a useful biomarker for assessing disease severity of COVID-19 pneumonia [14]. Indeed, in COVID-19 critically ill patients requiring mechanical ventilation or intensive therapy, KL-6 serum level is increased. This biomarker has already been used to evaluate the prognosis of various interstitial lung diseases (ILDs), including idiopathic pulmonary fibrosis (IPF) [15–17]. Recently, it has been proposed as useful tool to evaluate acute respiratory distress syndrome (ARDS) and infective pneumonia, like severe acute respiratory syndrome coronavirus 2 (SARS-CoV-2) [14].

Computed tomography (CT) has a high sensitivity in SARS-CoV-2 patients; it is widely used to aid patient management being the reference diagnostic modality in pulmonary involvement assessment and in patient stratification [18].

Some authors, such as Pan et al. and Francone et al., have proposed a very useful CT score for a semiquantitative assessment of disease extension [19, 20].

Moreover, there are significant correlations between the degree of lung inflammation and the main clinical symptoms and laboratory findings [21].

However, to our knowledge, there are not many scientific evidences of correlation between CT score and KL-6 value during the course of COVID-19 disease.

The primary objective of this retrospective study in COVID-19 patients is to validate the possible role of KL-6 as a biomarker of disease severity, comparing KL-6 levels with CT images based on the semiquantitative score.

## Methods

### Patients selection criteria

Between March 28, 2020, and May 21, 2020, we retrospectively enrolled 196 consecutive patients with strong suspicion of COVID-19 interstitial pneumonia presenting to our Emergency Unit. This single-center study was approved by the Ethics Committees of our Institute (protocol number CE 109/2020), and written informed consent was waived due to the retrospective nature of the study.

Patients with highly suspected COVID-19 pneumonia were included, in accordance with internal guideline criteria.

All subjects underwent reverse transcriptase polymerase chain reaction (RT—PCR) detection test for SARS-CoV-2, chest CT scan and blood test including KL-6, antibodies COVID-19 (Ab COVID-19 BAU/mL, creatinine (n.v. 0.70–120 mg/dL), Interleukin-6 (IL-6; n.v. 1.5–7.0 pg/mL), D-dimer (n.v. 50–420 ng/mL) and platelets count (n.v. 150–450 × 10^9^/l), prothrombin time (PT; n.v. 11–16 s) with derived measures as percent prothrombin activity (PT%; n.v. 70–120) and international normalized ratio (INR; n.v. 0.81–120).

After RT-PCR detection test for SARS-CoV-2, patients were divided into two groups: COVID-19-positive group (*N* = 162) and non-COVID-19 group (*N* = 34).

In subsequent analyses, to avoid changes in the inflammatory panel due to disease progression, we included only patients who underwent blood sampling for KL-6 within 5 days from CT scan.

The final population consisted of 63 patients, including 37 COVID-19-positive patients and 26 with negative RT-PCR testing for SARS-CoV-2.

**Fig. 1:**
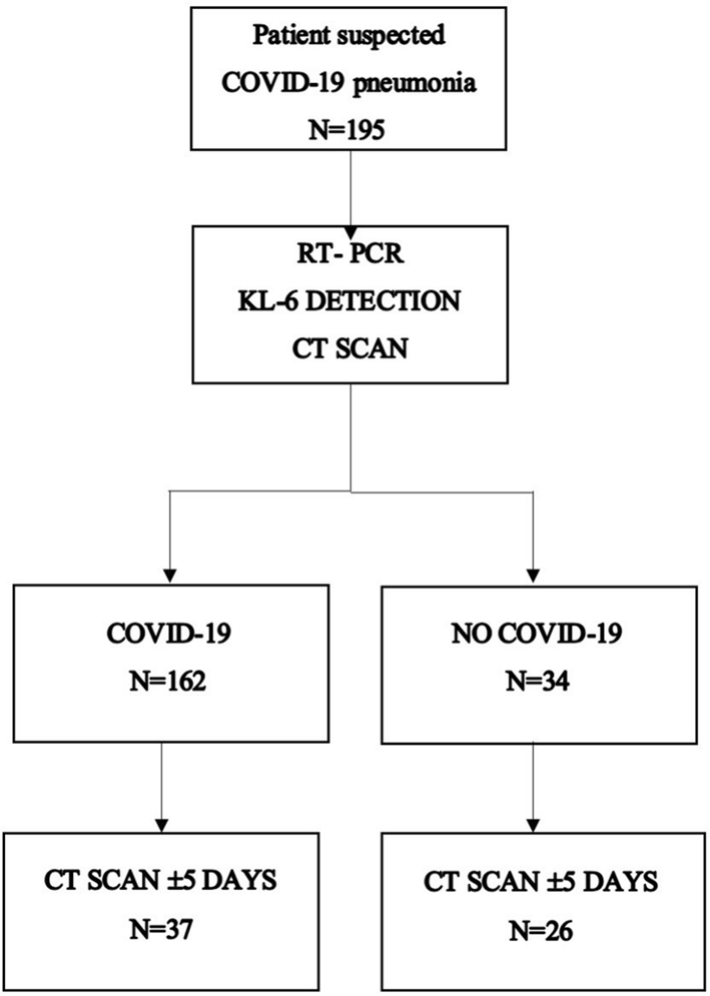
Flowchart of the patient search and selection process

The flowchart of the patient selection process is shown in Fig. 1.

### KL-6 and IL-6 detection

A blood sample was sent to the laboratory for the determination of KL-6 and IL-6 levels, at the first admission to the Emergency Department, before starting therapy.

KL-6 serum values were determined on a Lumipulse G1200 (Fujirebio-Europe, Gent, Belgium), using the LUMIPULSE G KL-6-II kit (Fujirebio, Tokyo, Japan). The principle of the method is agglutination of sialylated carbohydrate antigen in samples with KL-6 monoclonal antibody through antigen–antibody reaction. KL-6 detection range considered is between 50 and 10.000 U/mL, with intra-assay CV of < 4.4% and inter-assay CV of < 10%, and as a clinical cut-off we considered 400 U/mL. This value was established considering the mean value in a population of blood donors belonging to the transfusion center of our Institute, enrolled consecutively. We considered the value present at the 95th percentile on one hundred samples.

All standard biochemical analyses were performed by Elecysis Roche Instruments.

IL-6 detection range was between 1.5 and 5000 pg/mL; as a clinical cut-off, we considered 7.0 pg/mL.

### CT protocol and imaging analysis

A multidetector CT scanner (Somatom Sensation 64; Siemens Healthineers) was used for examinations. All patients were instructed in breath-holding.

The following acquisition parameters have been used: 120 kV, 100 mAs, pitch 1.5 and collimation 0.6 mm; all images were reconstructed with a slice thickness of 1.00 mm, 512 × 512 mm, with both a sharp and soft kernel. Coronal and sagittal multiplanar reconstructions were also available in all cases.

**Fig. 2:**
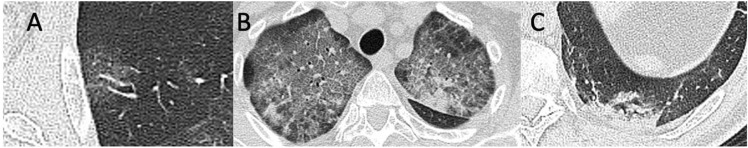
Chest CT findings of coronavirus disease 2019 (COVID-19): **a** ground glass opacity (GGO); **b** crazy-paving pattern (GGO and inter- and intralobular septal thickening); **c** consolidation

CT images were reviewed by two radiologists (L.M., P.R.) with at least 20-year experience in chest imaging, in consensus. For all patients, CT images were evaluated considering the presence of three thoracic CT patterns: (i) ground glass opacity, (ii) consolidation, (iii) crazy paving in agreement with the international standard nomenclature defined by the Fleischner Society glossary and previous literature on viral pneumonia [22–24][Fig. 2].

**Fig. 3:**
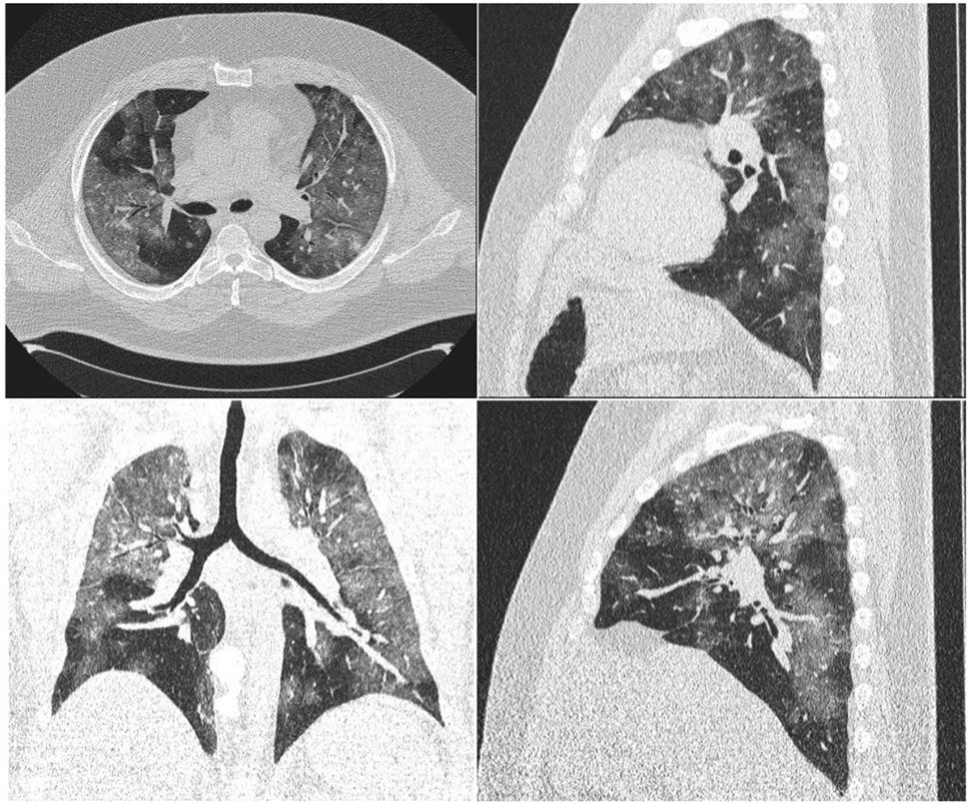
Example of a semiquantitative CT score in a 38-year-old with SarS-CoV-2 pneumonia and a global CT score value of 21

A semiquantitative evaluation of lung involvement was calculated for each of the 5 lobes according to Francone et al. [19] as follows: 0, no involvement; 1, < 5% involvement; 2, 5–25% involvement; 3, 26–50% involvement; 4, 51–75% involvement; and 5, > 75% involvement. The resulting global CT score was the sum of each individual lobar score (0 to 25) [Fig. 3].

Pleural effusion, fibrosis, single/multiple nodules with halo sign, lymphadenopathy, air bronchogram, bronchial thickening and bronchiectasis have also been reported.

### Statistical analysis

Spearman’s rho coefficient was used to evaluate the linear relationship between the CT score and the KL-6 value. Data are expressed as median and interquartile range (IQR) for continuous variables and frequencies for categorical ones. Comparison between two groups was performed by Chi-square test or Mann–Whitney *U* test as appropriate. Spearman’s correlation index was used to assess the relationship between continuous measurements. A logistic regression model was used to determine the possible value of CT score in predicting the risk of critical illness in COVID-19 patients. All tests were two-tailed, and a value of *P* < 0.05 was considered as statistically significant. Analyses were performed using R version 4.0.1 (The R Project for Statistical Computing).

## Results

### Population

The final population included *n* = 37 COVID-19-positive patients (18 male, 19 female; median age 67) and *n* = 26 patients (10 male, 16 female; median age 74.50) with a negative RT-PCR test for SARS-CoV-2. Baseline characteristics are summarized in Table 1. In the COVID-19 group, 64% had KL-6 values ​​ > 400 U/mL (24/37 patients). As regards the other serum proteins, we found a significant association between D-Dimer and COVID-19 patients. The median value of D-dimer in the COVID-19-positive group was 4409 ng/mL, whereas in the non-COVID19 control group it was 1116,50 ng/mL, with a statistically significant difference between the two groups (*p* = 0.004).

**Table 1:** Patients’ baseline characteristics. Unless otherwise noted, data are in median value, with interquartile range (IQR) in parenthesis

	COVID-19-positive group	Non-COVID-19 group	*p*-value
Gender*			
Male	18 (48.6)	10 (38.5)	0.587
Female	19 (51.4)	16 (61.5)	
Age [yrs]	67.00 (52.00–80.00)	74.50 (56.50–81.75)	0.402
CT score	5.00 (4.00–11.00)	3.50 (2.00–7.00)	0.086
KL-6 [U/mL]	2.93 (0.25–20.87)	0.09 (0.07–0.10)	** < 0.001**
Ab COVID-19 [BAU/mL]	2.93 (0.25–20.87)	0.09 (0.07–0.10)	** < 0.001**
Serum creatinine [mg/dL]	0.89 (0.71–1.03)	0.93 (0.66–1.25)	0.383
PT [s]	11.19 (10.72–11.58)	11.85 (11.22–13.25)	**0.010**
PT%	109.50 (102.50–117.50)	99.50 (78.25–109.50)	**0.014**
INR [s]	0.99 (0.94–1.04)	1.06 (0.99–1.19)	**0.010**
IL-6 [pg/mL]	24.71 (9.27–50.96)	28.74 (3.08–45.22)	0.784
D-dimer [ng/mL]	4409.00 (1024.00–4409.00)	1116.50 (569.00–2024.25)	**0.004**
Platelets	247.00 (206.75–287.00)	270.00 (210.00–309.50)	0.385
KL-6 [U/mL]*			0.928
≤ 400	24 (64.9)	18 (69.2)	
> 400	13 (35.1)	8 (30.8)	

*Numbers of patients, with percentages in parentheses

A value of *P*<0.05 was considered as statistically significant. Significant values are already in bold

### CT score and KL-6 protein

**Fig. 4:**
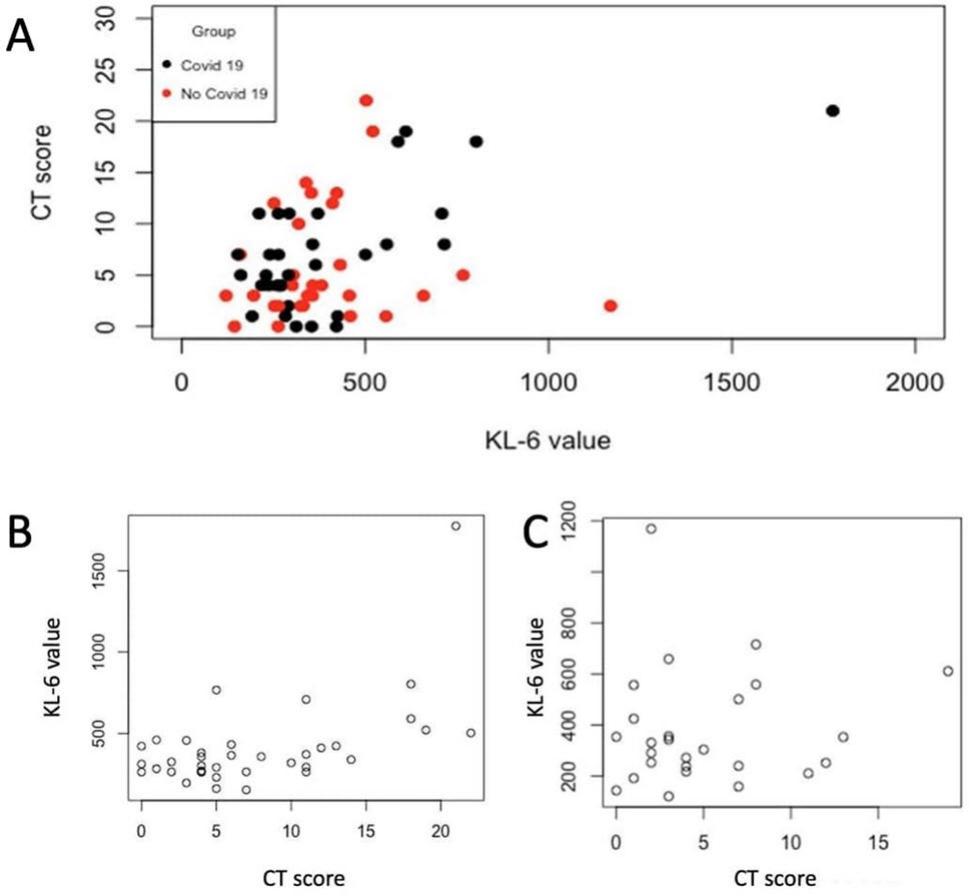
Comparisons between CT scores and the KL-6 value in both groups of patients. Red dots in only COVID-19 patients and black dots in only non-COVID-19 patients

CT score correlated with the KL-6 value in the whole population (rho = 27, *p*-value 0.035) (Fig. 4a). When considering only COVID-19-positive patients, this correlation was also present (rho = 0.423, *p*-value 0.009) (Fig. 4b). No statistically significant correlation was found between CT score and KL-6 value in the non-COVID-19 group alone (Fig. 4c).

**Fig. 5:**
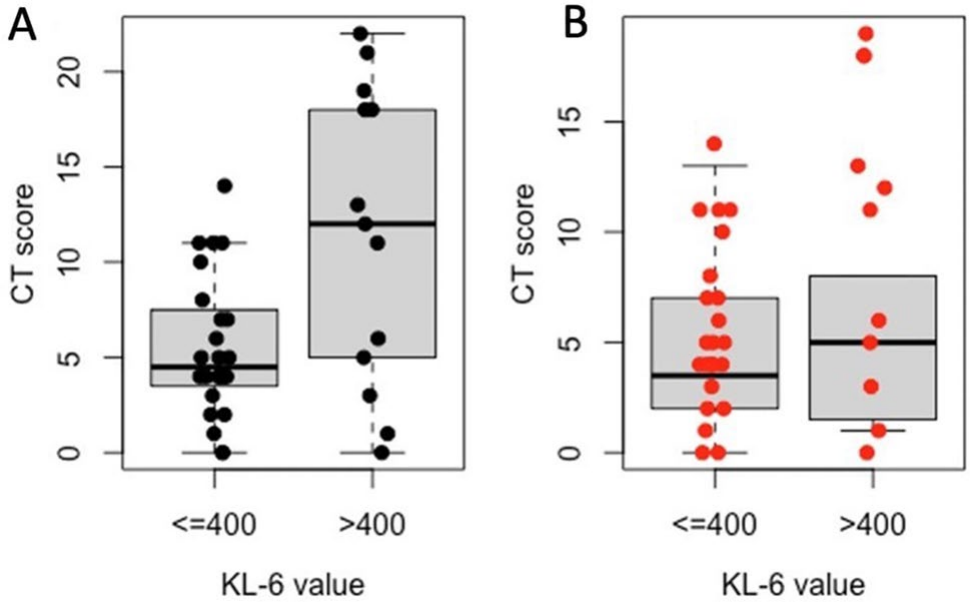
Box plot of CT score stratified by KL-6 ≤ 400 and KL-6 > 400. **a** COVID-19-positive patients, and **b** non-Covid-19 group. Lines in the box represent the median (the thickest line), 25th, and 75th percentiles (the thinnest lines, below and above the median, respectively), and the whiskers (error bars) below and above the box indicate the 10th and 90th percentiles, respectively

In COVID-19-positive group, CT score median value was significantly higher in patients with KL-6 > 400 U/mL than in patients KL-6 ≤ 400 U/mL (12.00, IQR 5.00–18.00 versus 4.50, IQR:3.75–7.25; *p*-value 0.027) (Fig. 5a). Also, in non-COVID-19 group CT score was higher in patients with KL-6 > 400 U/mL patients than in patients with KL-6 ≤ 400 U/mL patients, although this difference was not statistically significant (5.00, IQR:1.75–8.00 versus 3.50, IQR:2.00–6.50, *p*-value 0.716) (Fig. 5b).

### Imaging features

Concerning CT findings, we observed a significant difference in radiological patterns between patients with KL-6 ≤ 400 U/mL and KL-6 > 400 U/mL in COVID-19-positive group. In particular, in COVID-19-positive patients with KL-6 > 400 U/mL the significant radiological patterns were "crazy paving" at the right upper (*n* = 8; 61.5%) and middle lobe (*n* = 4; 30.8%) and "consolidation" at the middle lobe (*n* = 5; 38.5%) (Table 2).

**Table 2:** Comparison in positive Covid-19 patients stratified by the value of KL-6 about radiological main pattern and its localization

Radiological pattern	COVID-19-positive patients with KL-6 ≤ 400 U/mL (*n* = 24)	COVID-19-positive patients with KL-6 > 400 U/mL (*n* = 13)	*p*-value
*Ground glass* LUL LLL RUL ML RLL	21 (87.5) 19 (79.2) 15 (62.5) 13 (54.2) 9 (37.5) 13 (54.2)	12 (92.3) 10 (76.9) 9 (69.2) 8 (61.5) 8 (61.5) 9 (69.2)	1.000 1.000 0.961 0.933 0.291 0.589
*Crazy paving* LUL LLL RUL ML RLL	11 (45.8) 4 (16.7) 9 (37.5) 1 (4.2) 0 (0.0) 8 (33.3)	9 (69.2) 5 (38.5) 6 (46.2) 8 (61.5) 4 (30.8) 7 (53.8)	0.309 0.283 0.872 ** < 0.001** **0.020** 0.388
*Consolidation* LUL LLL RUL ML RLL	17 (70.8) 7 (29.2) 12 (50.0) 3 (12.5) 1 (4.2) 14 (58.3)	12 (92.3) 6 (46.2) 9 (69.2) 5 (38.5) 5 (38.5) 10 (76.9)	0.273 0.501 0.436 0.158 **0.025** 0.441

Unless otherwise noted, data are numbers of patients, with percentages in parentheses

*LUL* Left upper lobe, *LLL* left lower lobe; *RUL* right upper lobe; *ML* middle lobe; *RLL* right lower lobe

A value of *P*<0.05 was considered as statistically significant. Significant values are already in bold

## Discussion

Since the outbreak of the COVID-19 pandemic, several studies have been published on the role of inflammatory biomarkers to select different patient groups based on the impact of infection and prediction of outcome; however, these have often been nonspecific or in common with other diseases.

More recently, serum KL-6 has also been investigated and found to be a prognostic biomarker of COVID-19 disease, with increased values in more severely affected cases [17, 25].

Classifying the degree of disease considering not only clinical but also radiological or laboratory parameters is a crucial issue due to the lack of specific criteria. Several radiological studies highlight the primary role of CT in classifying disease severity, particularly in assessing the extent of parenchymal involvement by constructing CT scores, as suggested by some authors [19, 20].

In our study, we aimed to find a correlation between serum KL-6 protein and CT disease severity score by evaluating both a group of patients with Sars-Cov-2 and a control group with suggestive symptoms but negative RT-PCR test.

As a first result, we found a positive correlation between serum KL-6 values and CT score in the group of COVID-positive patients. Specifically, patients with higher values of the serum biomarker (> 400 U/ mL) were associated with a greater extent of parenchymal disease, with median CT score value of 12, whereas in patients with lower KL-6 values (< 400 U/mL) the median CT score value was 4.5 (*p*-value 0.027). Moreover, we also tested for a possible correlation between KL-6 values and CT score in the control group; however, we found no significant association in these patients (*p*-value 0.716).

A retrospective longitudinal analysis of 166 COVID-19 patients by Deng K. et al. showed significantly higher KL-6 values in severe/critical COVID-19 patients (*n* = 17) than in mild COVID-19 patients (*n* = 149) [26]. Moreover, in this study, the late value of serum KL-6 (+ 7 days; *N* = 80) was significantly associated with the presence of areas of lung injury on CT. In a subsequent paper, Varble N. al. sought to investigate and characterize the associations between clinical, laboratory, and imaging characteristics of asymptomatic and pre-symptomatic patients with SARS-CoV-2, finding that serum KL-6 value was one of the best parameters to distinguish asymptomatic patients with COVID-19-related CT infiltrates from asymptomatic patients without CT changes [27]. Finally, in more recent work by Arnold DT et al., the authors found that patients with abnormal CT scans at 12 weeks had significantly higher KL-6 levels during recovery than the group with negative CT [28].

In agreement with the above-mentioned works, we also found a significant association between KL-6 elevation and the presence of radiological alterations. However, differently from them, we also quantified the degree of CT lesion extension using a semiquantitative score, finding that increased KL-6 was associated not only with the presence but also with a greater extent of parenchymal lesions (CT score higher than 12).

As a second result, in our study we also found a significant difference in D-dimer values in the two populations, with higher values in the COVID-19 group (*p* = 0.004). This finding is well known in the literature, and our study is a further confirmation of the close relationship between COVID-19 infection, D-dimer value, and thus thrombotic risk, although the underlying mechanism needs further investigation [29, 30].

In our study, an interesting finding is a greater frequency of crazy paving pattern and consolidations involving the right upper and middle lobe in COVID-19-positive patients with KL-6 > 400 U/mL. According to some authors, these radiological findings may refer to a more severe and late state of disease [19]. The data confirm that a high KL-6 value can really correlate with a more severe state of the disease [31].

Our study has some limitations. First, it is a single-center study with a small cohort of patients. In fact, because patients referred to our unit were evaluated in emergency during a pandemic, not all clinical and laboratory data were always available.

Second, we did not make correlations between radiological and laboratory data with clinical outcomes; after initial admission to our emergency unit, the management of these patients was deferred to other departments or to the attending physician, so these data were not available for the entire cohort.

## Conclusion

Our preliminary findings showed that the dynamic profile of KL-6 in COVID-19-positive patients correlates closely with CT lung severity, as determined by a semiquantitative score.

Further studies with larger populations and validation with clinical outcomes are needed to confirm our results, but serum KL-6 value at baseline may represent an early biomarker of lung disease extension in patients with Sars-Cov2 infection.

## Data Availability

The datasets analyzed during this study are available from the corresponding author upon reasonable request. Not applicable.

## Abbreviations

COVID-19: Coronavirus disease 2019
SARS-CoV-2: Severe acute respiratory syndrome coronavirus 2
DIC: Disseminated intravascular coagulation
KL-6: Krebs von den Lungen-6
ILDs: Interstitial lung diseases
IPF: Idiopathic pulmonary fibrosis
PT: Prothrombin time
INR: International normalized ratio
IL-6: Interleukin-6
CT: Computer tomography
CARDS: COVID-19-related acute respiratory distress syndrome
IQR: Interquartile Range
RT-PCR: Retrotrascriptase polymerase chain reaction
